# Correlation between n-3 polyunsaturated fatty acids consumption and BDNF peripheral levels in adolescents

**DOI:** 10.1186/1476-511X-13-44

**Published:** 2014-03-05

**Authors:** Charles Francisco Ferreira, Juliana Rombaldi Bernardi, Vera Lúcia Bosa, Ilaine Schuch, Marcelo Zubaran Goldani, Flávio Kapczinski, Giovanni Abrahão Salum, Carla Dalmaz, Gisele Gus Manfro, Patrícia Pelufo Silveira

**Affiliations:** 1 Post Graduate Program in Neuroscience, Institute of Basic Sciences/Health, Federal University of Rio Grande do Sul (UFRGS), Porto Alegre, RS, Brazil; 2 Laboratory of Stress Neurobiology, Biochemistry Department, Federal University of Rio Grande do Sul (UFRGS), Porto Alegre, RS, Brazil; 3 Center for Child and Adolescent Health Studies (NESCA), Translational Pediatrics Laboratory (LPT), Hospital de Clínicas de Porto Alegre (HCPA), Faculty of Medicine, Federal University of Rio Grande do Sul (UFRGS), Porto Alegre, RS, Brazil; 4 Anxiety Disorders Outpatient Program for Children and Adolescents, National Institute of Developmental Psychiatry for Children and Adolescents (INPD, CNPq), Graduate Program in Medical Sciences: Psychiatry, Hospital de Clínicas de Porto Alegre (HCPA), Faculty of Medicine, Federal University of Rio Grande do Sul (UFRGS), Porto Alegre, RS, Brazil; 5 Laboratory of Molecular Psychiatry, Psychiatry Department, Hospital de Clínicas de Porto Alegre (HCPA), Schoool of Medicine, Federal University of Rio Grande do Sul (UFRGS), Porto Alegre, RS, Brazil; 6 Rua Ramiro Barcelos 2600 Anexo, Departamento de Bioquímica, UFRGS. CEP 90035–000, Porto Alegre, RS, Brazil

**Keywords:** Anxiety, Children, Adolescents, SCARED, BDNF

## Abstract

**Background:**

Although several studies have reported an association between mental disorders and serum levels of brain-derived neurotrophic factor (BDNF), this association is still poorly understood. The study of factors associated with both BDNF levels and mental disorders, such as n-3 polyunsaturated fatty acids (n-3 PUFAs), may help to elucidate the mechanisms mediating the relationship between the two variables. Therefore, the present study aimed to evaluate whether the intake n-3 PUFAs correlates with serum levels of BDNF.

**Findings:**

This study involved 137 adolescents drawn from a community sample, including a group with high levels of anxiety, assessed using the Screen for Children and Anxiety Related Emotional Disorders. Blood samples were collected and serum BDNF levels were measured. n-3 PUFAs were estimated using a food frequency questionnaire for adolescents. Correlations were performed to assess the association between n-3 PUFAs intake and BDNF levels. Effects of potential confounders (total fat consumption, age, gender and anxiety) were examined using linear regression models. There was a direct correlation between n-3 PUFAs consumption and serum BDNF levels, which remained significant even after accounting for potential confounders.

**Conclusions:**

We were able to detect a correlation between n-3 PUFAs intake and peripheral BDNF levels. Our study was limited by its small sample size, and our external validity may be restricted by the oversampling of anxious adolescents. Our findings may help determine the nature of the association between mental disorders and serum levels of BDNF. However, more studies are needed to elucidate the possible mechanisms by which n-3 PUFAs intake affects BDNF levels, and how this may lead to an increased vulnerability to psychiatric disorders.

## Background

Brain-Derived Neurotrophic Factor (BDNF) is a dimeric protein thought to be involved in neuronal survival and synaptic plasticity, and to be an important biomarker for psychiatric conditions such as depression and bipolar disorder [1-3]. It is a member of the growth factor family and acts as a regulator of synaptic plasticity, synaptogenesis, as well as neuronal survival and differentiation [4-6]. Therefore, the understanding of processes associated with both BDNF levels and mental disorders may ultimately help determine the underlying mechanisms responsible for the association between these two variables.

Studies of human subjects have demonstrated that lower per capita fish/seafood consumption, which can be used as a surrogate measurement of n-3 polyunsaturated fatty acids (PUFAs) dietary intake, is associated with a higher prevalence of bipolar spectrum disorders [7,8], major depression [9,10] and postpartum depression [11,12]. Experimental studies in rats demonstrate that polyunsaturated fatty acids (PUFAs) could modify brain BDNF levels [13-15] and may play an important role in the function and structure of many membrane proteins [16,17]. Nevertheless, there is still a need to investigate whether the relationship between dietary n-3 PUFAs intake and BDNF translates to humans. In light of these above findings, the aim of the present study was to evaluate whether the consumption of n-3 PUFAs correlates with serum BDNF levels in humans.

## Findings

The current study involved 137 adolescents drawn from a community sample, including a group with high rates of anxiety (64.6% female; mean age 13.9 years, SD = 2.42), as assessed by the Screen for Children and Anxiety Related Emotional Disorders (SCARED) [18]. A detailed description of the sampling process can be found elsewhere [19]. Briefly, all quartiles of the distribution of SCARED scores were equally represented in the sample (i.e., no to very low anxiety, mild anxiety, moderate anxiety and severe anxiety), although there was an oversampling of participants in the upper quartile (severe anxiety). The SCARED inventory comprises 38 items that can be grouped into subscales according to the different anxiety symptoms investigated, and consists of a screening tool for DMS-IV childhood anxiety disorder. The present study was approved by the Research Ethics Committee of the Hospital de Clínicas de Porto Alegre (GPPG/HCPA, protocol number 08–481). Informed consent was provided by all primary caretakers, and all adolescents assented to participate in the study.

Blood samples were collected between 7 and 10 am after a fasting period of 10–12 hours, centrifuged for 5 minutes at 4500 rpm, and serum was stored at −80°C in order to measure BDNF levels. All BDNF measurements were performed on the same day by sandwich-ELISA using monoclonal antibodies specific for BDNF (R&D Systems, Minneapolis, Minnesota), according to the manufacturer’s instructions. The intra-assay and inter-assay coefficients of variation were 3.7 and 8.5%, respectively. n-3 PUFAs intake was estimated by a food frequency questionnaire administered to the adolescents (FFQ) [20]. The quantitative analysis of macro and micronutrient intake was performed using the NutriBase® software (Version NB7 Network) [Phoenix, AZ, USD]. Spearman correlations were performed to assess the association between n-3 PUFA intake and BDNF levels. The effects of potential confounders (total fat intake, age, gender and symptoms of anxiety) were examined using linear regression models (involving log-transformed BDNF values, logBDNF).

**Figure 1:**
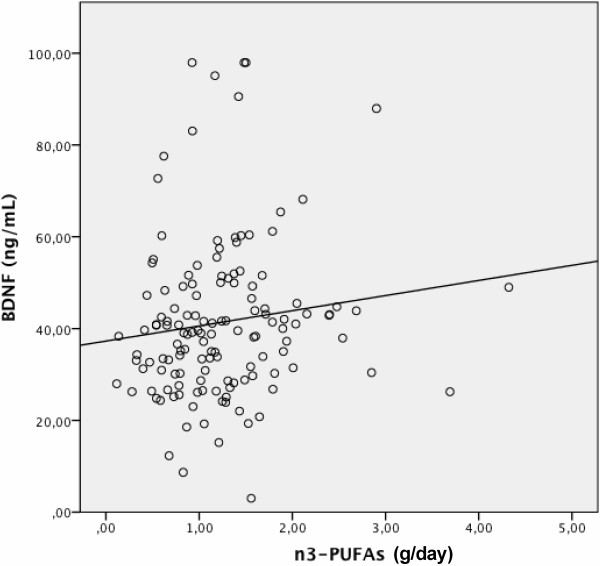
**Correlation between n-3 polyunsaturated fatty acids (n-3 PUFAs) intake (g/day) and peripheral levels (ng/mL) of brain-derived neurotrophic factor (BDNF).** The correlation between n-3 PUFAs intake and BDNF levels was statistically significant (r_s_ = 0.172, *p* = 0.043).

The correlation between n-3 PUFA intake and BDNF levels was statistically significant, r_s_ = 0.172, *p* = 0.043 (Figure 1). Multivariate linear regression models showed that even after controlling for the confounders, n-3 PUFA intake was associated with higher levels of logBDNF (beta = .463, t = 2.83, *p* = 0.005). No evidence of collinearity was found in the model.

## Discussion

We were able to show that n-3 PUFAs intake is associated with serum BDNF levels in adolescents, corroborating to the suggestion that the relationship between mental disorders and BDNF may be mediated by the intake of nutrients such as n-3 PUFAs. This may influence the cellular and physiological processes involved in psychiatric/mood disorders, such as the fatty acid composition of cellular membranes, the modulation of behavioral systems, neurodevelopment, and oxidative stress in specific brain areas [21-25].

Studies suggest that n-3 PUFA-depleted rodents exhibit changes in emotional status such as elevated aggression, depression and anxiety [26-31]. Moreover, the dietary deprivation of n-3 PUFAs in rats may lead to changes in the expression of BDNF in the frontal cortex, in cAMP response element binding protein (CREB) activity and reduce p38 mitogen-activated protein kinase (MAPK) activity [13].

It is noteworthy that the food frequency questionnaire used in the study contained a varied list of 94 foods or drinks [20,32]. Thus, the corresponding food sources to the n-3 PUFAs consumption among students were diverse, including vegetable oils and products/preparations based in oils (e.g. biscuits, mayonnaise and fried foods), according to calculations performed with the values depicted in the American Food Table [33]. Besides fish and oils consumption, red meat is also considered a source of n-3 PUFAs [34], and our region is especially known to have a high consumption of meat. A national survey conducted in both urban and rural areas throughout the country with analysis of individual food consumption in Brazil from 2008–2009 [35], reported that the Southern region of Brazil, including the State of Rio Grande do Sul, where the city Porto Alegre lays, was identified as one of the leading regions in the consumption of red or pork meat. Besides, it was also observed that among 10 to 13 years old population, the n-3 PUFAs displayed a high daily consumption (mean: 1.5 g) in this region, compared to other Brazilian regions. Meat intake is traditionally high and typical from our region, which could probably corroborate to the high n-3 PUFAs consumption levels evidenced in this study.

It is important to note that our study is limited by its small sample size, and that the oversampling of anxious adolescents may restrict our external validity. However, in spite of these limitations, we were able to detect a correlation between n-3 PUFAs consumption and serum BDNF levels. The extent to which peripheral BDNF levels are representative of central nervous system BDNF levels remains to be investigated, although positive correlations between serum and cortical BDNF levels have been reported in preclinical studies [36], and cellular and behavioral brain functions have been found to be modulated by peripheral BDNF [37]. Furthermore, serum BDNF levels have been associated with neuronal integrity in healthy subjects [38] and decreased BDNF peripheral levels (i.e. serum or plasma) have been consistently found in patients with mood disorders [39,40]. BDNF is implicated in a variety of neural processes and is related to neuronal developmental stages in both animals and humans [39]. Lang, Hellweg, Sseifert, Schubert and Gallinat, 2007 [38] reported that serum BDNF protein concentrarion might reflect some aspects of neuronal plasticity in the living human brain, playing a role as regulators of neuronal survival and differentiation. Similar findings have been reported by Poo, 2001 [6]. Recent studies demonstrate that decreased peripheral BDNF levels are consistently related to disease activity and progression in bipolar disorder [41] as well as to the cognitive impairment seen in patients following their first psychotic episode [42]. More studies are needed to elucidate the possible mechanisms by which n-3 PUFAs consumption affects BDNF levels and influences the vulnerability to psychiatric and mood disorders, especially during certain life stages such as adolescence.

## Abbreviations

BDNF: Brain-derived neurotrophic factor; DMS IV: Diagnostic and statistical manual of mental disorders IV; ELISA: Enzyme-linked immunosorbent assay; n-3 PUFAs: Omega-3 polyunsaturated fatty acids; PUFAs: Polyunsaturated fatty acids; Rpm: Revolutions per minute; SCARED scale: Screen for children and anxiety related emotional disorders scale.

## Competing interest

Charles Francisco Ferreira is a CAPES scholarship recipient and declares no potential conflicts of interest. Giovanni Abrahão Salum has a CAPES/FAPERGS post-doctoral scholarship and declares no potential conflicts of interest. Vera Lúcia Bosa and Ilaine Schuch declare no potential conflicts of interest. Flávio Kapczinski, Patrícia Pelufo Silveira, Carla Dalmaz, Gisele Gus Manfro and Marcelo Zubaran Goldani have received research grants from Brazilian government institutions (CNPQ, FAPERGS and FIPE-HCPA).

## Authors’ contributions

All authors participated in the study to a significant extent. CFF, JRB, GAS, CD, GGM, PPS worked on data analysis and interpretation, in writing the manuscript and contributed to the intellectual content of the article. VLB, IS, MZG, FK, GAS, GGM and PPS worked on study conception and design, as well as data collection and interpretation, and made intellectual contributions to the article. All authors read and approved the submitted manuscript.

## References

[B1] Shimizu E Hashimoto K Okamura N Koike K Komatsu N Kumakiri C Nakazato M Watanabe H Shinoda N Okada S Iyo M Alterations of serum levels of brain-derived neurotrophic factor (BDNF) in depressed patients with or without antidepressants Biol Psychiatry 2003 54 70 75 10.1016/S0006-3223(03)00181-1 12842310

[B2] Kapczinski F Frey BN Kauer-Sant'Anna M Grassi-Oliveira R Brain-derived neurotrophic factor and neuroplasticity in bipolar disorder Expert Rev Neurother 2008 8 1101 1113 10.1586/14737175.8.7.1101 18590480

[B3] Kauer-Sant'Anna M Kapczinski F Andreazza AC Bond DJ Lam RW Young LT Yatham LN Brain-derived neurotrophic factor and inflammatory markers in patients with early- vs. late-stage bipolar disorder Int J Neuropsychopharmacol 2009 12 447 458 10.1017/S1461145708009310 18771602

[B4] Park H Poo MM Neurotrophin regulation of neural circuit development and function Nat Rev Neurosci 2013 14 7 23 10.1038/nrc3653 23254191

[B5] Manji HK Drevets WC Charney DS The cellular neurobiology of depression Nat Med 2001 7 541 547 10.1038/87865 11329053

[B6] Poo MM Neurotrophins as synaptic modulators Nat Rev Neurosci 2001 2 24 32 10.1038/35049004 11253356

[B7] Noaghiul S Hibbeln JR Cross-national comparisons of seafood consumption and rates of bipolar disorders Am J Psychiatry 2003 160 2222 2227 10.1176/appi.ajp.160.12.2222 14638594

[B8] McNamara RK Evaluation of docosahexaenoic acid deficiency as a preventable risk factor for recurrent affective disorders: current status, future directions, and dietary recommendations Prostaglandins Leukot Essent Fatty Acids 2009 81 223 231 10.1016/j.plefa.2009.05.017 19515544

[B9] Hibbeln JR Fish consumption and major depression Lancet 1998 351 1213 9643729 10.1016/S0140-6736(05)79168-6

[B10] De Vriese SR Christophe AB Maes M Lowered serum n-3 polyunsaturated fatty acid (PUFA) levels predict the occurrence of postpartum depression: further evidence that lowered n-PUFAs are related to major depression Life Sci 2003 73 3181 3187 10.1016/j.lfs.2003.02.001 14561523

[B11] Shapiro GD Fraser WD Séguin JR Emerging risk factors for postpartum depression: serotonin transporter genotype and omega-3 fatty acid status Can J Psychiatry 2012 57 704 712 23149286 10.1177/070674371205701108 PMC5173356

[B12] da Rocha CM Kac G High dietary ratio of omega-6 to omega-3 polyunsaturated acids during pregnancy and prevalence of post-partum depression Matern Child Nutr 2012 8 36 48 10.1111/j.1740-8709.2010.00256.x 22136220 PMC6860680

[B13] Rao JS Ertley RN Lee HJ DeMar JC Arnold JT Rapoport SI Bazinet RP n-3 polyunsaturated fatty acid deprivation in rats decreases frontal cortex BDNF via a p38 MAPK-dependent mechanism Mol Psychiatry 2007 12 36 46 10.1038/sj.mp.4001888 16983391

[B14] Vetrivel U Ravichandran SB Kuppan K Mohanlal J Das UN Narayanasamy A Agonistic effect of polyunsaturated fatty acids (PUFAs) and its metabolites on brain-derived neurotrophic factor (BDNF) through molecular docking simulation Lipids Health Dis 2012 11 109 10.1186/1476-511X-11-109 22943296 PMC3477081

[B15] Ferreira CF Bernardi JR Krolow R Arcego DM Fries GR de Aguiar BW Senter G Kapczinski FP Silveira PP Dalmaz C Vulnerability to dietary n-3 polyunsaturated fatty acid deficiency after exposure to early stress in rats Pharmacol Biochem Behav 2013 107 11 19 23537731 10.1016/j.pbb.2013.03.006

[B16] Youdim KA Martin A Joseph JA Essential fatty acids and the brain: possible health implications Int J Dev Neurosci 2000 18 383 399 10.1016/S0736-5748(00)00013-7 10817922

[B17] Spector AA Yorek MA Membrane lipid composition and cellular function J Lipid Res 1985 26 1015 1035 3906008

[B18] Isolan L Salum GA Osowski AT Amaro E Manfro GG Psychometric properties of the Screen for Child Anxiety Related Emotional Disorders (SCARED) in Brazilian children and adolescents J Anxiety Disord 2011 25 741 748 10.1016/j.janxdis.2011.03.015 21514788

[B19] Salum GA Isolan LR Bosa VL Tocchetto AG Teche SP Schuch I Costa JR Costa Mde A Jarros RB Mansur MA Knijnik D Silva EA Kieling C Oliveira MH Medeiros E Bortoluzzi A Toazza R Blaya C Leistner-Segal S Salles JF Silveira PP Goldani MZ Heldt E Manfro GG The multidimensional evaluation and treatment of anxiety in children and adolescents: rationale, design, methods and preliminary findings Rev Bras Psiquiatr 2011 33 181 195 10.1590/S1516-44462011000200015 21829913

[B20] Willett WC Nutritional Epidemiology 1998 Oxford: Oxford University Press

[B21] Balanzá-Martínez V Fries GR Colpo GD Silveira PP Portella AK Tabarés-Seisdedos R Kapczinski F Therapeutic use of omega-3 fatty acids in bipolar disorder Expert Rev Neurother 2011 11 1029 1047 10.1586/ern.11.42 21721919

[B22] Liperoti R Landi F Fusco O Bernabei R Onder G Omega-3 polyunsaturated fatty acids and depression: a review of the evidence Curr Pharm Des 2009 15 4165 4172 10.2174/138161209789909683 20041818

[B23] Rombaldi Bernardi J de Souza ER Ferreira CF Pelufo Silveira P Fetal and neonatal levels of omega-3: effects on neurodevelopment, nutrition, and growth ScientificWorldJournal 2012 2012 202473 23125553 10.1100/2012/202473 PMC3483668

[B24] Tur JA Bibiloni MM Sureda A Pons A Dietary sources of omega 3 fatty acids: public health risks and benefits Br J Nutr 2012 107 Suppl 2 S23 S52 22591897 10.1017/S0007114512001456

[B25] Williams LL Kiecolt-Glaser JK Horrocks LA Hillhouse JT Glaser R Quantitative association between altered plasma esterified omega-6 fatty acid proportions and psychological stress Prostaglandins Leukot Essent Fatty Acids 1992 47 165 170 10.1016/0952-3278(92)90155-C 1461929

[B26] Zimmer L Hembert S Durand G Breton P Guilloteau D Besnard JC Chalon S Chronic n-3 polyunsaturated fatty acid diet-deficiency acts on dopamine metabolism in the rat frontal cortex: a microdialysis study Neurosci Lett 1998 240 177 181 10.1016/S0304-3940(97)00938-5 9502233

[B27] Fedorova I Salem N Jr Omega-3 fatty acids and rodent behavior Prostaglandins Leukot Essent Fatty Acids 2006 75 271 289 10.1016/j.plefa.2006.07.006 16973342

[B28] Zimmer L Delion-Vancassel S Durand G Guilloteau D Bodard S Besnard JC Chalon S Modification of dopamine neurotransmission in the nucleus accumbens of rats deficient in n-3 polyunsaturated fatty acids J Lipid Res 2000 41 32 40 10627499

[B29] Zimmer L Vancassel S Cantagrel S Breton P Delamanche S Guilloteau D Durand G Chalon S The dopamine mesocorticolimbic pathway is affected by deficiency in n-3 polyunsaturated fatty acids Am J Clin Nutr 2002 75 662 667 11916751 10.1093/ajcn/75.4.662

[B30] Mathieu G Oualian C Denis I Lavialle M Gisquet-Verrier P Vancassel S Dietary n-3 polyunsaturated fatty acid deprivation together with early maternal separation increases anxiety and vulnerability to stress in adult rats Prostaglandins Leukot Essent Fatty Acids 2011 85 129 136 10.1016/j.plefa.2011.07.001 21784625

[B31] Mathieu G Denis S Lavialle M Vancassel S Synergistic effects of stress and omega-3 fatty acid deprivation on emotional response and brain lipid composition in adult rats Prostaglandins Leukot Essent Fatty Acids 2008 78 391 401 10.1016/j.plefa.2008.05.003 18579362

[B32] Slater B Philippi ST Fisberg RM Latorre MR Validation of a semi-quantitative adolescent food frequency questionnaire applied at a public school in São Paulo, Brazil Eur J Clin Nutr 2003 57 629 635 10.1038/sj.ejcn.1601588 12771963

[B33] USDA National Nutrient Database for Standard Reference, Release 25 2012

[B34] Howe PR Meyer BJ Record S Baghurst K Contribution of red meat to very long chain omega-3 fatty acid (VLCOmega3) intake Asia Pac J Clin Nutr 2003 12 S2715023629

[B35] IBGE Pesquisa de Orçamentos Familiares 2008–2009, Análise de Consumo Alimentar no Brasil 2011 2011: Instituto Brasileiro de Geografia e Estatística (IBGE)

[B36] Karege F Schwald M Cisse M Postnatal developmental profile of brain-derived neurotrophic factor in rat brain and platelets Neurosci Lett 2002 328 261 264 10.1016/S0304-3940(02)00529-3 12147321

[B37] Schmidt HD Duman RS Peripheral BDNF produces antidepressant-like effects in cellular and behavioral models Neuropsychopharmacology 2010 35 2378 2391 10.1038/npp.2010.114 20686454 PMC2955759

[B38] Lang UE Hellweg R Seifert F Schubert F Gallinat J Correlation between serum brain-derived neurotrophic factor level and an in vivo marker of cortical integrity Biol Psychiatry 2007 62 530 535 10.1016/j.biopsych.2007.01.002 17560556

[B39] Frey BN Andreazza AC Houenou J Jamain S Goldstein BI Frye MA Leboyer M Berk M Malhi GS Lopez-Jaramillo C Taylor VH Dodd S Frangou S Hall GB Fernandes BS Kauer-Sant'Anna M Yatham LN Kapczinski F Young LT Biomarkers in bipolar disorder: a positional paper from the International Society for Bipolar Disorders Biomarkers Task Force Aust N Z J Psychiatry 2013 47 4 321 332 10.1177/0004867413478217 23411094

[B40] Grande I Kapczinski F Stertz L Colpo GD Kunz M Cereser KM Kauer-Sant'Anna M Frey B Vieta E Magalhaes PV Peripheral brain-derived neurotrophic factor changes along treatment with extended release quetiapine during acute mood episodes: an open-label trial in drug-free patients with bipolar disorder J Psychiatr Res 2012 46 1511 1514 10.1016/j.jpsychires.2012.08.017 22939945

[B41] Fernandes BS Gama CS Ceresér KM Yatham LN Fries GR Colpo G de Lucena D Kunz M Gomes FA Kapczinski F Brain-derived neurotrophic factor as a state-marker of mood episodes in bipolar disorders: a systematic review and meta-regression analysis J Psychiatr Res 2011 45 995 1004 10.1016/j.jpsychires.2011.03.002 21550050

[B42] Ruiz de Azua S Matute C Stertz L Mosquera F Palomino A de la Rosa I Barbeito S Vega P Kapczinski F González-Pinto A Plasma brain-derived neurotrophic factor levels, learning capacity and cognition in patients with first episode psychosis BMC Psychiatry 2013 13 27 10.1186/1471-244X-13-27 23320462 PMC3567944

